# Promoting children’s pain alleviation in emergency department

**DOI:** 10.1186/1824-7288-40-S1-A53

**Published:** 2014-08-11

**Authors:** BM Cantoni

**Affiliations:** 1 SITRA Area Assistenziale della Pediatria Fondazione IRCCS Ca’ Granda Ospedale Maggiore Policlinico, 20122 Milan, Italy

## Background

Pain is, by nature, a subjective experience influenced by social, psychological, and experiential factors.

Nowadays, pain relief is considered an imperative of the pediatric emergency nursing. Analgesia administration influences the entire child’s medical experience and can have a lasting effect on patient and family’s reaction to current and future medical cares [1].

Pain assessment represents the first step for analgesia management and it should be performed routinely by nurses in emergency department (ED) triage along with vital signs monitoring. Increasing evidence indicates that prompt pain evaluation improves the whole quality of subsequent care. Findings from studies investigating the use of scales for pain evaluation, in addition to the experience gained in clinical practice, suggest to treat all patients presenting a pain intensity major than 4 [2] (scale’s range from 0 – no pain to 10 – the worst pain experienced). However, the myth that analgesic treatments would mask symptoms and delay diagnosis in some cases (i.e. children with acute abdominal disease) still frightens physicians and nurses. Therefore, pain reduction is often not considered as a primary task to be accomplished by the ED team [3].

## Materials and methods

We retrospectively analyzed data regarding admission causes, pain assessment (Numeric Rate Scale, Face, Legs, Activity, Cry, Consolability scale) and management of 23677 children admitted to the pediatric ED of the “Ca’ Granda Ospedale Maggiore Hospital in Milan between January and December 2013.

## Results

The first reason requiring medical attention was fever (37%). Pain was the second cause (25%). Moreover, children affected by other disease reported concomitant pain in 42% of cases.

Pain was assessed and recorded in 85% of children. A pain with an intensity major than 4 was detected in 31% of cases. Only 72% were treated with an analgesic drug. The non-treated children suffered from abdominal pain. Acetaminophen was the main drug administered (15 mg/kg/dose).

## Conclusions

Our data indicate that pain is an important cause of concern for families that leads to medical consultation. However, pain assessment at triage is not performed in all patients, yet. Although several data show that analgesia does not mask signs of acute abdominal diseases, nurses still avoid the prompttreatment of abdominal pain. Although non-pharmacological interventions, such as distraction, positioning, sucrose, have been described to be useful in reducing abdominal pain [4] they are often unsatisfactory. In conclusion, we suggest that educational interventions and staff trainings should be addressed to promote children’s pain alleviation at triage.
